# Accurate identification of oxygen desaturation status in COPD by using classifier ensemble

**DOI:** 10.1371/journal.pone.0318837

**Published:** 2025-02-05

**Authors:** Yue-Fang Wu, Xin Shu, Shiqi Wang, Xiaojun Xu, Pei-Li Sun

**Affiliations:** 1 Department of Internal Medicine, Nanjing University of Science and Technology Hospital, Nanjing, Jiangsu, China; 2 School of Computer Science, Jiangsu University of Science and Technology, Zhenjiang, Jiangsu, China; 3 Department of Respiratory Medicine, The First Affiliated Hospital with Nanjing Medical University, Nanjing, Jiangsu, China; Khalifa University of Science and Technology, UNITED ARAB EMIRATES

## Abstract

The accurate identification of oxygen desaturation (OD) status plays critical role in the clinic diagnosis of chronic obstructive pulmonary disease (COPD), which is a common disease related to the lungs and respiratory tract of the human body. This paper focuses on a specific type of OD status, i.e., exercise-induced oxygen desaturation (EIOD) status in COPD, and try to further improve the performance of EIOD status identification. We propose a new and effective EIOD status identification method by using classifier ensemble strategy. In the proposed method, five different features of each data point from the time series of SpO2 and pulse are extracted and then combined to form the discriminative feature of the corresponding data point; then, multiple base classifiers with different balanced training subsets are trained and then integrated by using *AdaBoost* Algorithm. The comparative computational results on the 6-min walk test (6MWT) of the recruited participants show that the proposed method achieved the best global performance with *AUC* (Area Under Curve) value of 0.8532, indicating that the proposed method can be effectively used for the identification of EIOD and could assist the clinic diagnosis of COPD.

## Introduction

Chronic Obstructive Pulmonary Disease (COPD) is a common chronic disease related to the lungs and respiratory tract of the human body [1, 2]. The characteristic of COPD is the persistent airflow limitation and progressive development [3]. Although COPD is a disease that can be prevented and treated, it seriously endangers the physical and mental health of patients [1, 4]. Previous medical research has shown that COPD is a disease with high incidence rate and mortality worldwide, which causes serious economic and social burden, and this burden is still increasing [5, 6]. The earlier COPD is diagnosed, the more effective it is to prevent and treat it early. Therefore, improving the level of clinic diagnosis and treatment of COPD is of great significance and in urgent need for improving people’s health.

Oxygen desaturation (OD) specifically refers to a significant decrease in the patient’s saturation of pulse oxygen (SpO2) [7, 8]. Among different kinds of OD, exercise-induced oxygen desaturation (EIOD) is common in COPD and routinely assessed during the 6-min walk test (6MWT) [9]. Various definitions have been used to characterize significant EIOD. In this study, we focus on the identification of EIOD and use the most common definition of EIOD as a pulse oxygen saturation (SpO2) drop > 4% from baseline and/or a lowest SpO2 < 90% during the 6MWT [10, 11].

In clinical practice, the EIOD is one of the key criteria for diagnosing COPD [8]: the medical staff usually first try to obtain the personal data such as the saturation of pulse oxygen (SpO2) and pulse rate (PR) of the patient over a period of time; then, the medical staff try to manually label out the potential data points that are in EIOD from the SpO2 curve by analyzing the characteristics of the patient’s SpO2 and PR curves; Finally, the medical staff will determine whether the patient has COPD or signs of COPD according to their own clinical experiences.

However, there are two obvious shortcomings in manually determining EIOD, which will be further used to diagnose COPD, through above-mentioned manual methods: the first disadvantage is that these manual methods are often time-consuming because the medical staff will have to collect data from devices and perform necessary labeling and calibration works; the second one is the issue of accuracy of EIOD determination, which heavily depends on the clinical experiences of medical staff [7, 8, 12]. Developing effective EIOD status identification model, which can automatically label out the potential data points that are in EIOD status, is in urgent need. In view of this, we try to design and implement an EIOD status identification model by using classifier ensemble technique in this study. Once the identification model is trained on the collected training dataset, it can be directly used to automatically identify the potential data points that are in EIOD status for those unseen data from new patients.

During the past decades, artificial intelligence technologies represented by machine learning have been widely applied to the medical fields and great achievements have been made [13–16]. Hence, we believe that the above-mentioned two disadvantages of the manual methods for COPD diagnosis could be well relieved if machine learning techniques could be appropriately introduced into the diagnosis of COPD, leading to the significant improvements of the efficiency and accuracy for COPD diagnosis. However, it is found that there is still limited research in developing machine learning based methods for the diagnosis of COPD. In view of this, together with the fact that EIOD is one of the key criteria for clinically diagnosing COPD, we recently have carried out preliminary research on machine learning based EIOD status identification. In reference [7], we proposed a method, termed as AttLSTM, for the identification of EIOD in COPD by using attention-based long short-term memory neural network (LSTM). In AttLSTM, four discriminative features, including SpO2 value, PR value, window feature of SpO2, and gradient feature of SpO2, are extracted from each data point (in EIOD or non- EIOD) on SpO2 and PR time series and then combined to form the discriminative feature vector for that data point; then, an AttLSTM model is trained for the identification of EIOD based on the feature vectors of all the collected data points. It is shown that AttLSTM model reaches the *AUC* (area under curve) value of 0.8531 on testing dataset. To further improve the performance of EIOD status identification, we incorporated another new feature component, i.e., correlation feature of SpO2, into the four feature components in AttLSTM; further, we replaced AttLSTM model with supervised self-organizing map model (SSOM) [12]. Computational experimental results show that the global performance index, i.e., *AUC*, is further improved from 0.8531 to 0.8611, indicating that new discriminative feature and powerful machine learning model can further enhance the performance of EIOD status identification [12].

Although progress has been made, there still has room for the improvement of performance in EIOD identification. In this study, we focus on EIOD and aim to further improve the performance of EIOD status identification to facilitate the subsequent clinic COPD diagnosis by taking classifier ensemble strategy: First, five different feature components, which have been demonstrated useful for EIOD status identification [7, 12], are extracted and combined to form the discriminative feature of each data point; Then, we train multiple base classifiers, each of which is trained on a balanced training subset, to form a classifier pool; After that, an ensembled classifier is obtained from the trained classifier pool by using *AdaBoost* algorithm; Finally, the ensembled classifier can be used for the EIOD status identification. Comparative experimental results demonstrate the superiority of the proposed method over other existing approaches for EIOD status identification. Detailed data analyses show that the advanced performance of the proposed method is mainly attributed to the ensemble strategy that can effectively utilize the complementarity of identification results from the underlying base classifiers. We believed that the proposed EIOD status identification method will provide important reference value for the clinic diagnosis of COPD.

## Materials and methods

### Data collection and calibration

High quality datasets play a crucial role in training high-performance and highly generalizable machine learning models. In this study, we used the data described in reference [8]: The First Affiliated Hospital of Nanjing Medical University and Nanjing University of Technology Hospital collaboratively conducted raw data collection and calibration work from Dec 29, 2016 to Dec 28, 2017: a total of 85 volunteers were recruited, including 69 COPD patients and 16 normal individuals.

The 6-min walk test (6MWT) conducted in this study is a routine clinical examination item, and the pulse oxygen detector (pulse oximeter wristwatch) is also a non-invasive detection instrument used in clinical practice. We fully informed the volunteers of the testing process, precautions, and related risks during the trial, and obtained their informed verbal consents. We also assured volunteers that all collected data will be encrypted and stored to ensure the security of medical data and the protection of personal privacy. In addition, all collected data must undergo desensitization before being used for research, including this study. After review by the ethics committee of The First Affiliated Hospital of Nanjing Medical University (ethical review number: 2015-SR-207.A1), this study is exempt from obtaining informed written consent of volunteers.

By wearing a pulse oximeter wristwatch (model: ORANGER, CMS50S) on the wrists of each volunteer, we collected their six-minutes’ walk data mainly including two attributes: saturation of pulse oxygen (SpO2) and pulse rate (PR). The original raw data of each volunteer is a matrix which consists of six columns, i.e., Timestamp (timestamp), SpO2 value (blood oxygen saturation index, scalar), PR value (pulse rate, scalar), IsValid (indicating whether the current data is valid, True or False), Quality (indicating the quality of the current data, True or False), IsEvent (indicating whether the corresponding data point is a candidate EIOD point, True or False), and IsEIOD (indicating whether the current data point is a true EIOD point, True or False). A data point (being labeled as IsEIOD = 1) is valid and make sense if and only if IsValid = True, Quality = True, and IsEvent = True, while a data point (being labeled as IsEIOD = 0) is valid and make sense if and only if IsValid = True, Quality = True, and IsEvent = False. For the collected raw data, it is first manually calibrated by senior medical staff to filter and remove abnormal data points. After the screening and calibration steps, the six-minutes’ walk data of each volunteer can be simplified into a four-column matrix, denoted as, with each column having the following meanings: Timestamp, SpO2 (saturation of pulse oxygen) value, PR (pulse rate) value, and IsEIOD (indicating whether the corresponding data point is in an exercise-induced oxygen desaturation (EIOD) status: 1 indicates in EIOD status; 0 indicates in non-EIOD status).

Each volunteer completes the 6-minute walking test in a 30 meters long corridor and the detailed 6MWT data collection protocol is as follows:

Step 1: Make sure that the volunteer has no contraindications to the walking test (i.e., ruling out the possibility of danger to the volunteer from the walking test);
Step 2: The volunteer wears a pulse oximeter wristwatch (model: ORANGER, CMS50S) with a finger cuff on the middle finger, and ensures that the wristwatch functions properly.
Step 3: Measure the blood pressure and the blood oxygen saturation of the volunteer;
Step 4: The volunteer makes effort to complete the 6-minute walking test. The wristwatch will automatically records data and related symptoms. Note that in this phase, the volunteer is allowed to slow down when chest tightness and asthma symptoms appear;
Step 5: Observe the data from the volunteer’s wristwatch and remove the wristwatch until the blood oxygen saturation returns to the value of 95 or the value before the 6-minute walking test.

### Feature encoding

As described in the Section of Data Collection and Calibration, the six-minutes’ walk data of each participant is a four-column matrix. To facilitate the subsequent description, we remove the first column (Timestamp) and obtain a three-column matrix as shown in Eq (1), where *s*_*i*_, *p*_*i*_ and *l*_*i*_ in the *i*-th line of *M* are the SpO2 value, the PR value and the EIOD label of the *i*-th data point:

$$M=(\begin{array}{ccc} \mathrm{SPO}2 & PR & \mathrm{IsEIOD}\\ s_{1} & p_{1} & l_{1}\\ s_{2} & p_{2} & l_{2}\\ \vdots & \vdots & \vdots \\ s_{i} & p_{i} & l_{i}\\ \vdots & \vdots & \vdots \\ s_{n} & p_{n} & l_{n} \end{array})$$ (1)

For the *i*-th data point, the most simple and intuitive feature vector can be formulated as:

$$f_{i}={\left(s_{i},p_{i}\right)}^{T}$$ (2)

However, previous studies have shown that whether a data point in EIOD status depends not only on the SpO2 and PR values at that time, but also on the SpO2 and PR values of its neighboring data points. In view of this, Hu [8] extended ***f***_*i*_ by adding the widow feature ($s_{i}^{window}$) and gradient feature ($s_{i}^{gradient}$) of SpO2 as shown in Eq (3):

$$f_{i}={\left(s_{i},s_{i}^{window},s_{i}^{gradient},p_{i}\right)}^{T}$$ (3)

where $s_{i}^{window}$ is the window feature of the *i*-th data point obtained by averaging the SpO2 values in the window centered at the *i*-th data point; $s_{i}^{gradient}$, as defined in Eq (4), is the gradient feature of SpO2 of the *i*-th data point:

$$s_{i}^{gradient}=\frac{s_{i}-s_{i-1}}{s_{i-1}}$$ (4)

Further, Wu *et al*. [12] further improved the discriminability of the feature vector $f_{i}={\left(s_{i},s_{i}^{window},s_{i}^{gradient},p_{i}\right)}^{T}$ by incorporating a new feature component (correlation coefficient) and obtained the new feature vector as defined in Eq (5):

$$f_{i}^{extended}={\left(s_{i},s_{i}^{window},s_{i}^{gradient},p_{i},s_{i}^{correlation}\right)}^{T}$$ (5)

where $s_{i}^{correlation}$ is the correlation coefficient feature component as defined in Eq (6):

$$s_{i}^{correlation}={\left(\theta_{i}^{1},\theta_{i}^{2},\cdots ,\theta_{i}^{G}\right)}^{T}$$ (6)

where $\theta_{i}^{g}=\frac{1}{w_{size}-g}\sum_{t=i-\frac{w_{size}-1}{2}}^{i+\frac{w_{size}-1}{2}-g}{\left(s_{t}-s_{t+g}\right)}^{2}$, 1≤*g*≤*G*, *w*_*size*_ is the size of the window centered at the *i*-th data point, and *G* is a prescribed integer.

Considering the good discriminability of the feature $f_{i}^{extended}={\left(s_{i},s_{i}^{window},s_{i}^{gradient},p_{i},s_{i}^{correlation}\right)}^{T}$, in this study, we also use this feature to represent each data point in the dataset.

### Dealing with imbalance by using classifier ensemble

**Fig 1:**
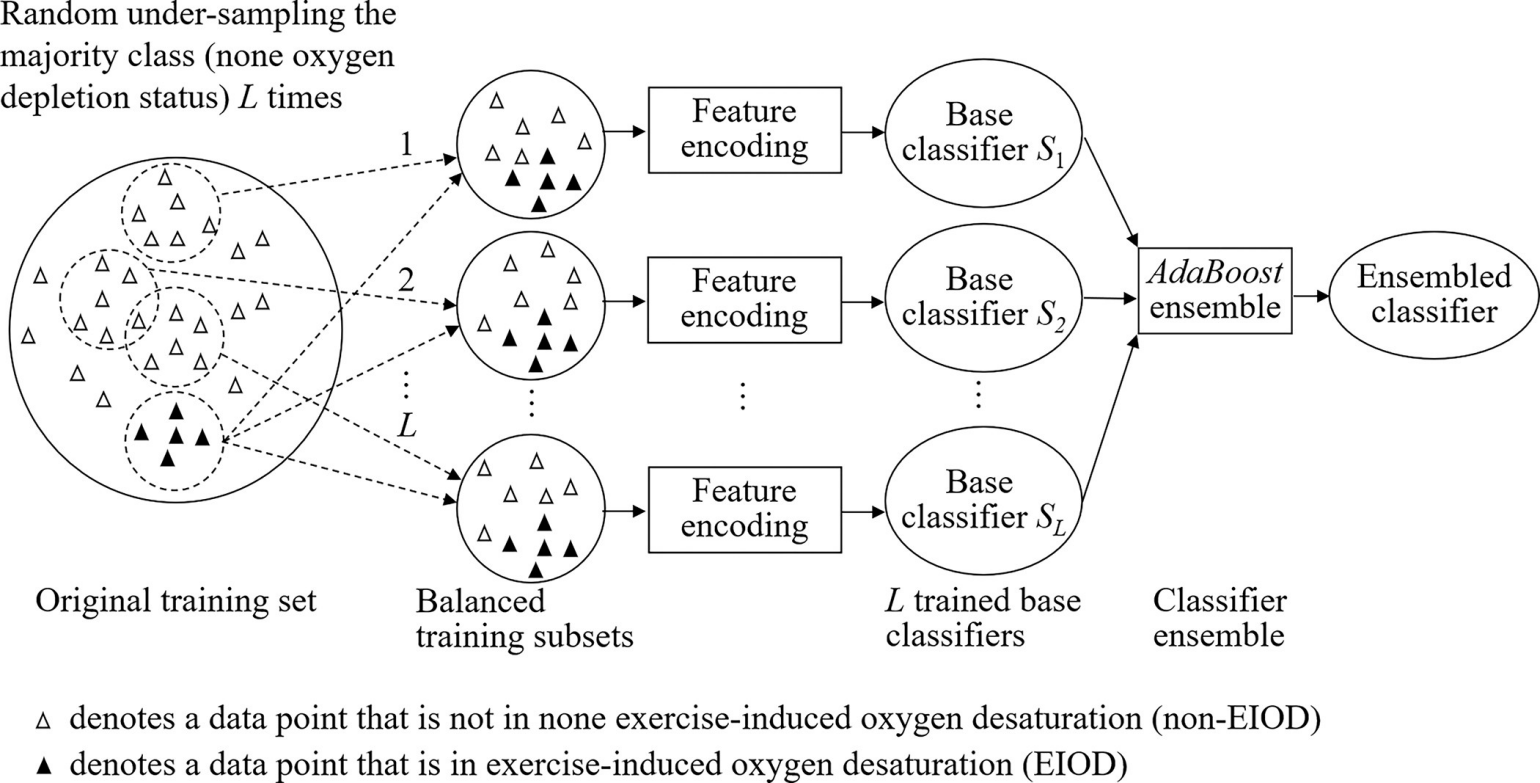
Workflow of the *AdaBoost* classifier ensemble based on random under-sampling technique for exercise-induced oxygen desaturation (EIOD) identification.

By carefully analyzing the data points in the calibrated dataset, it is found that the number of positive data points (in EIOD) is far less than that of the negative data points (in non-EIOD). In other words, the machine learning based EIOD status identification is a typical imbalanced problem, where the number of samples in majority class (non-EIOD) is significantly larger that of samples in minority class (EIOD). Previous studies have demonstrated that directly applying the machine learning algorithms, which assume that samples in different classes are balanced, to imbalanced problems tends to a poor performance [17]. To deal with this problem, we adopted the workflow, as shown in Fig 1, which ensembles multiple base classifiers trained on balanced training subsets with random under-sampling techniques [18]. More specifically, we sample *L* different majority training subsets, each of which consists of the same number of samples to that of the minority class, by randomly under-sampling the majority class *L* times; then, we train a base classifier on each of the sampled majority training subset plus the minority training set; finally, the *L* trained based classifiers are ensembled by using *AdaBoost* [19, 20] to form the final ensembled classifier.

Let *x* be the feature vector encoded by using Eq (6) of an input sample (i.e., data point in this study), *Pool* = {*S*_1_(***x***),*S*_2_(***x***),⋯,*S*_*i*_(***x***),⋯,*S*_*L*_(***x***)} be the set of trained base classifier pool, the *AdaBoost* ensemble is to develop the classifier team $S^{E}=\{S_{1}^{E}(x),S_{2}^{E}(x),\cdots ,S_{k}^{E}(x),\cdots ,S_{K}^{E}(x)\}$, *K*≤*L* and $S_{k}^{E}(x)\in Pool$, by incrementally selecting one base classifier each time from the pool *S*. The ensembled classifier is $S(x)=\sum_{k=1}^{K}\alpha_{k}S_{k}^{E}(x)$, where *α*_*k*_ is the weight of the selected base classifier $S_{k}^{E}(x)$. Finally, the ensembled classifier *S*(***x***) can be used to perform identification for those unseen samples. Details for *AdaBoost* algorithm could be found in [19] or [20].

### Training and testing procedures

We conducted all the experiments by taking leave-one-out cross-validation (LOOCV) strategy on the calibrated dataset: we take 1 people’s data to construct the testing set and the remaining 84 peoples’ data were used to construct the training set; this practice continued until all the 85 peoples’ data were traversed over. In each round of the LOOCV, we obtain *L* balanced training subsets by using random under-sampling technique from the training set; then, for each data point in all the *L* training subsets, we encoded it in to a feature vector as defined in Eq (5); after that, *L* learning models can be trained on the *L* training subsets, respectively, with extracted feature vectors; finally, the *L* trained models are ensembled by using *AdaBoost* [19, 20] to form the final ensembled classifier. For those data points in the testing set, each of which will be first encoded into a feature vector as defined in Eq (5) and then fed into the final ensembled classifier. The outputs of the final ensembled classifier for all the data points will be used for calculating the evaluation indices.

### Evaluation indices

The trained ensembled classifier *S*(***x***) can then be used for the identification of EIOD for those unseen data points as follows: First, the feature vector (i.e.,***x***) of an unseen data point is extracted and fed to the trained ensembled classifier; Then, the corresponding output of the trained ensembled classifier, denoted as *p*, is the probability of being in EIOD, the range of which is 0~1; Finally, we determine whether the unseen data point is of being in EIOD by using a prescribed threshold *T*: the unseen data point is in EIOD if the predicted probability *p* is greater than *T*; otherwise, the unseen data point is in non-EIOD. By varying the value of *T* from 0 to 1, we can obtain the Receiver Operating Characteristic (*ROC*) Curve of the trained ensembled classifier. The area under the *ROC* Curve, denoted as *AUC*, is a global performance index of the trained ensembled classifier [17]. The larger the value of *AUC*, the higher the global performance of the trained ensembled classifier. In view of this, we will use *AUC* as the global performance index to evaluate the performance the proposed and the compared classifiers.

## Experiments and analysis

### Experimental configuration and parameter setting

To objectively evaluate the performance of the proposed method and fairly compare it with other existing EIOD status identification methods, we performed comparative experiments of all considered methods on the same experimental configuration as follows:

1. Each experiment was performed by using leave-one-out cross-validation (LOOCV) strategy as described in the Section of Training and Testing Procedures;
2. Each data point was encoded into a feature vector $f_{i}^{extended}={\left(s_{i},s_{i}^{window},s_{i}^{gradient},p_{i},s_{i}^{correlation}\right)}^{T}$ as defined in Eq (5);
3. Three types of base classifiers, i.e., MLP [21], SVM [22] and LSTM [23] were considered to perform classifier ensemble for EIOD status identification.

It has not escaped from our notice that the discriminative capability of the extracted feature is closely related to the parameter setting, i.e., the window size (*w*_*size*_) and the correlation coefficient (*G*). Nevertheless, the optimal values of *w*_*size*_ and *G* depends not only on the dataset itself, but also on the base classifier used. In view of this, for the given calibrated dataset and a given base classifier (e.g., MLP, SVM and LSTM in this study), we obtained the optimal values of the two parameters by using a grid search strategy. More specifically, we vary the value of *w*_*size*_ from 5 to 15 and the value of *G* from 1 to 9, both with a step size of 2. For each parameter pair (*w*_*size*_,*G*), we tested the performance of the identification model trained with the given base classifier by taking leave-one-out cross-validation (LOOCV) strategy on the calibrated dataset; the parameter pair $\left(w_{size}^{*},G^{*}\right)$ which achieves the highest identification performance measured by *AUC* is selected as the best parameter set. By using this grid search strategy, for example, we found the optimal values of *w*_*size*_ and *G* are 11 and 5, respectively, under taking LSTM as the base classifier.

In the classifier ensemble stage, we set the size of the base classifier pool to be 11. The parameter *K*, i.e., the number of base classifiers used in the ensembled classifier, which depends on the type of base classifiers (MLP, SVM, and LSTM) used and is automatically determined by *AdaBoost* algorithm. In this study, it is found that the optimal value for parameter *K* is 7 under taking LSTM as the base classifier.

### Classifier ensemble helps to improve EIOD status identification

In this section, three popular classifiers, i.e., MLP [21], SVM [22] and LSTM [23] were used as base classifiers to demonstrate the efficacy of classifier ensemble for EIOD status identification. More specifically, for each type of base classifier (e.g., MLP), we first trained a single MLP identification model on the EIOD training set; then, we tested it and evaluated its performance on the EIOD testing set; after that, we further trained a corresponding ensembled classifier (e.g., Ensembled MLP) using the ensemble method as described in the Section of Dealing with Imbalance by Using Classifier Ensemble. Table 1 summarized the performance comparisons between different base classifiers and the corresponding ensembled classifiers for EIOD status identification. Note that for each type of base classifier and its corresponding ensembled classifier, both $f_{i}={\left(s_{i},s_{i}^{window},s_{i}^{gradient},p_{i}\right)}^{T}$ and $f_{i}^{extended}={\left(s_{i},s_{i}^{window},s_{i}^{gradient},p_{i},s_{i}^{correlation}\right)}^{T}$ were used as input feature vectors.

**Table 1:** Performance comparisons between different base classifiers and the corresponding ensembled classifiers for EIOD status identification.

Method	*AUC* ^*a*^	*AUC* ^*b*^
MLP	0.7829	0.7936
Ensembled MLP	0.7982	0.8152
SVM	0.8022	0.8103
Ensembled MLP	0.8186	0.8357
LSTM	0.8247	0.8314
Ensembled LSTM	0.8403	0.8532

^*a*^ $f_{i}={\left(s_{i},s_{i}^{window},s_{i}^{gradient},p_{i}\right)}^{T}$ as input feature vector

^*b*^ $f_{i}^{extended}={\left(s_{i},s_{i}^{window},s_{i}^{gradient},p_{i},s_{i}^{correlation}\right)}^{T}$ as input feature vector.

From Table 1, several observations can be made as follows: First, the discriminative capability of the feature vector $f_{i}^{extended}$ is superior to that of the feature vector ***f***_*i*_. As shown in Table 1, the *AUC*s of Ensembled MLP, Ensembled SVM and Ensembled LSTM under feature vector ***f***_*i*_ are 0.7982, 0.8186 and 0.8403, which are improved to 0.8152, 0.8357 and 0.8532 under feature vector $f_{i}^{extended}$. Similar phenomenon was also observed for the three base classifiers. This observation is consistent with conclusion in reference [12]. Second, for all the three base classifiers, their corresponding ensembled classifiers perform much better under both ***f***_*i*_ and $f_{i}^{extended}$ feature vectors. For example, compared with MLP, SVM and LSTM, the improvements of 2.7%, 3.1% and 2.6% were obtained by ensembled MLP, ensembled SVM and ensembled LSTM under feature vector $f_{i}^{extended}$. Also, the improvements of 2.0%, 2.0% and 1.9% for MLP, SVM and LSTM, respectively, were observed under feature vector ***f***_*i*_. This observation demonstrates that the ensembled classifier can well deal with the severe class imbalance in EIOD status identification problem thus can obtain better performance.

### Ablation experiments

In this section, we will investigate the contributions of different features to the EIOD status identification under ensembled LSTM model using LOOCV strategy. More specifically, we use (*s*_*i*_,*p*_*i*_) as baseline feature vector; then we gradually added the widow feature ($s_{i}^{window}$), the gradient feature ($s_{i}^{gradient}$) and the correlation coefficient feature ($s_{i}^{correlation}$) into the baseline feature vector. Table 2 summarizes the EIOD status identification performances with different feature combinations under ensembled LSTM model.

**Table 2:** Performance comparisons between different features under ensembled LSTM model using LOOCV strategy.

Feature	*AUC*
(*s*_*i*_,*p*_*i*_)	0.7713
$\left(s_{i},s_{i}^{window},p_{i}\right)$	0.8165
$\left(s_{i},s_{i}^{gradient},p_{i}\right)$	0.8247
$\left(s_{i},s_{i}^{window},s_{i}^{gradient},p_{i}\right)$	0.8403
$\left(s_{i},s_{i}^{window},s_{i}^{gradient},p_{i},s_{i}^{correlation}\right)$	0.8532

Several observations can be made by carefully analyzing Table 2. First, it is found that baseline feature, i.e., (*s*_*i*_,*p*_*i*_) achieved the *AUC* of 0.7713, demonstrating the possibility of performing EIOD status identification by directly using SpO2 and PR values; Second, improvements of 5.9% and 6.9% were observed by adding the widow feature ($s_{i}^{window}$) and the gradient feature ($s_{i}^{gradient}$), respectively, into the baseline feature vector. This observation demonstrates that the widow feature and the gradient feature have positive impacts to the identification of EIOD; Third, we found that when both $s_{i}^{window}$ and $s_{i}^{gradient}$ were added into the baseline feature vector, the value of *AUC* was further improved to 0.8403; Finally, it is found that the value of *AUC* can be further improved to the maximum value of 0.8532 after further adding the correlation coefficient feature component ($s_{i}^{correlation}$). The results in ablation experiments shown in Table 2 indicate that the gradient feature, the window feature, and the correlation coefficient feature are effective auxiliary features for identifying EIOD; By combining these three features with the baseline feature, the model’s capability for the identification of EIOD can be further significantly improved.

### Comparisons with existing EIOD status identification models

In this section, we compare the proposed ensembled LSTM with several state-of-the-art EIOD status identification models under the same feature representation method and on the same dataset. The first is the AttLSTM [7], which performs EIOD status identification by using an attention-based Long Short-Term Memory network; the second is SSOM [12], which identifies EIOD using a supervised self-organizing map neural network. Table 3 summarizes the performance comparisons of the proposed ensembled LSTM with AttLSTM and SSOM.

**Table 3:** Performance comparisons of the proposed ensembled LSTM with AttLSTM and SSOM.

Method	*AUC* ^*a*^	*AUC* ^*b*^
AttLSTM	0.8287	0.8306
SSOM	0.8314	0.8378
Ensembled LSTM	0.8403	0.8532

^*a*^ $f_{i}={\left(s_{i},s_{i}^{window},s_{i}^{gradient},p_{i}\right)}^{T}$ as input feature vector

^*b*^ $f_{i}^{extended}={\left(s_{i},s_{i}^{window},s_{i}^{gradient},p_{i},s_{i}^{correlation}\right)}^{T}$ as input feature vector.

By observing Table 3, it is found that the proposed ensembled LSTM achieved the best performance on EIOD status identification with *AUC*s of 0.8403 and 0.8532 under ***f***_*i*_ and $f_{i}^{extended}$ as input feature vectors, respectively. Compared with AttLSTM and SSOM, improvements of 2.7% and 1.8%, respectively, on *AUC* were obtained by ensembled LSTM under $f_{i}^{extended}$ input vector. Although not significant, the ensembled LSTM still achieved competitive and even better performance if compared with AttLSTM and SSOM under ***f***_*i*_. These superior performances under both ***f***_*i*_ and $f_{i}^{extended}$ over the two state-of-the-art EIOD status identification models further demonstrate the efficacy of the proposed ensembled LSTM.

## Conclusions

EIOD is one of the key criteria for diagnosing chronic obstructive pulmonary disease. The accurate identification of the EIOD for patients can help improve the diagnostic level of COPD and has important clinical significance. In order to reduce the workload of medical staff and further improve the timeliness and accuracy of EIOD status identification, this paper proposes an ensembled method for the identification of EIOD in COPD. In this method, we first extract five features of each data point from the time series of SpO2 and pulse; then, the extracted five features are serially combined to form the discriminative feature of the corresponding data point; Based on this feature representation, we train multiple base classifiers with different balanced training subsets and then integrate these trained base classifiers into an ensembled classifiers by using *AdaBoost* Algorithm. We tested three different base classifiers, i.e., MLP, SVM and LSTM. It is found that the ensembled LSTM performs best, which achieved the best *AUC* value of 0.8532. We also compared the ensembled LSTM with other two state-of-the-art EIOD status identification models, i.e., AttLSTM and SSOM, demonstrating its performance superiority for EIOD status identification in COPD.

Although the proposed method has achieved good performance on the EIOD status identification, it has not escaped from our notice that several issues deserve to be further investigated. First, the size of the dataset used in current study is relatively small, where only 69 COPD patients and 16 normal individuals are recruited. To further improve the generalization capability, we will collect more data to train the EIOD status identification model in future; Second, with the accumulation of EIOD data, we can develop more effective deep learning algorithms to further improve the EIOD status identification capability, thereby assisting the clinical diagnosis of COPD. Finally, we will extend the proposed method to the identification of other types of oxygen desaturation (e.g., sleep oxygen desaturation).
